# Distinguishable DNA methylation defines disease susceptibility influenced by race and ethnicity

**DOI:** 10.1186/s13148-021-01180-9

**Published:** 2021-10-11

**Authors:** Jairo A. Pinzon Cortes, Assam El-Osta

**Affiliations:** Epigenetics in Human Health and Disease Laboratory, Department of Diabetes, Central Clinical School, Monash University, Melbourne, VIC 3004 Australia

This editorial refers to: “Race/Ethnicity-Associated Blood DNA Methylation Differences Between Japanese and European American Women: An Exploratory Study”, by M-A Song et al., https://doi.org/10.1186/s13148-021-01171-w.

Ancestral differences exist between populations—and when it comes to the study of genetic and epigenetic variations, they are also misinterpreted. Instead of focussing on uniqueness among populations, many genetic consortiums look for commonalities between groups. In the contemporary age of genetics, the field is shifting towards providing insights of singularity based on ancestry, and none more relevant than the validation of biomarkers implicated with the progression of human disease. In actuality biomarkers of high specificity and sensitivity are a clinical requirement when providing pre-emptive therapy to those diagnosed of being at risk. The necessity of identifying unique traits remains underscored when considering disease stratification, which are often extrapolated from different ancestral populations. In this issue of *Clinical Epigenetics*, scientists in search of methylation indices have identified differences between Japanese and European American women that may be informative for disease progression (CLEP-D-21-00295 official citation).

This exploratory study was a nested cohort comprising 60 women, 30 Japanese Americans and 30 European Americans, derived from the MultiEthnic Cohort (MEC) from the same region of Oahu, Hawaii. A population study, the MEC, was originally conceived to assess relationships between diet and cancer in the USA that involve five ancestral backgrounds in Hawaii and California, namely African Americans, Latinos, Japanese Americans, Native Hawaiians and European Americans [1]. The authors sampled this population of women because of the elevated risk of liver disease [2] and NAFLD-associated hepatocellular carcinoma among Asian Americans [3].

In this issue of *Clinical Epigenetics*, the authors hypothesize that DNA methylation could be a signature that distinguishes Japanese women from European Americans. They postulate that DNA methylation could represent physiological differences in liver function and pathological conditions that are often associated with lifestyle changes including socio-economic disparities.

Japanese Americans had significantly higher blood CG methylation when compared to European Americans. The authors reported 174 differentially methylated CG sites with the majority (76%) of these hypermethylated in the Japanese Americans when compared to European counterparts. Differential methylation was predominantly found in the promoter regions of several genes implicated with liver function and disease such as tumour cell proliferation (*AFAP1* and *CSMD3*) and cirrhosis (*KIAA0748*/*TESPA1*). The authors of the study found other differentially methylated CG regions in relation to gene expression changes associated with pathophysiological processes such as adipogenesis and insulin signalling (*SOX6* and *SH3BP4*) and inflammation or fibrosis (*MIP1B/CCL4*). The authors hypothesize that these results could explain the higher susceptibility to liver diseases and cancer in Japanese Americans.

There are limitations with exploratory research of this type. Not only was the power of the study influenced by sample size, however, by virtue of study design they could not provide conclusions between sexes. While the study observed corresponding DNA methylation changes   using The Cancer Genome Atlas (TCGA) Research Network [4], the replication of blood derived differentially methylated genes with liver biopsies and disease outcomes awaits experimental validation. Other limitations acknowledged in the study were the cross-sectional use of the subset samples and possible confounders such as diet or other lifestyle factors. Despite the limitations, this and studies like it open new doors to integrative epigenetics and clinical interpretation to consider genetic ancestory , lifestyle and socio-economic influences. Take, for example, the assessments of disease risk associated with the ancestral background for African Americans and European Americans with advanced/end-stage heart failure [5]. That study identified distinguishable methylation indices from cardiac biopsies following procedures associated with the surgical placement of left ventricular assist device among men. Indeed, racial and socio-economic influences were associated with DNA methylation and end-stage heart failure. The unexpected findings also raise important questions of whether cardiac DNA methylation could be considered a distinguishable epigenetic determinant of socio-economic disparity and clinical diversity in vulnerable populations. Interpret this as you wish. The inclusion of race in clinical decision-making is controversial, facing criticism and scrutiny of study design and clinical endpoints.

Critically important—yet often difficult to achieve in studies such as those described above—are the considerations of diversity and ancestry as study design elements to improve clinical outcomes. Scientists are indeed shifting towards less abstract and favouring more informative descriptors—that have previously relied heavily on definitions by social constructs—advancing with guidelines that highlight forefront genomics [6]. While it is clear that categorizing people based on ethnicity is a social construct, we are also learning that differences in genetic ancestry could explain why some people are more susceptible to disease than others. And with respect to large consortium studies, there are more obvious similarities regarding global communities and critical reference populations. Many of the large genetic studies emphasize populations derived from European ancestry. For example, the genome aggregation database (gnomAD) that accounts for more than 50% of clinically relevant sites is from European populations [7]. Furthermore, genome-wide association studies report 78.39% of individuals are of European ancestry [8] and this also rings true for the genotype tissue expression (GTEx) project with 85.2% of participants from European origin [9]. Clearly, the rationale behind the exclusion of diverse populations in genetics research is multifactorial and it is acknowledged that this is also complicated.

While we appreciate that for antecedent cohorts this still remains problematic and without turnkey solutions essentially because they have not been widely adopted, there are in fact carefully considered and even nuanced solutions that engage scientists more directly than might be previously considered. With no obvious place to begin, the ADWG (ancestry and diversity working group https://clinicalgenome.org/working-groups/ancestry/) offers guideposts of consideration and information for scientists in what works. Despite these added responsibilities, there are the added benefits to study design and data collection that makes new space for clinical decision making. . In the modern genetics age, its early days for human epigenetics to break down these barriers—rather this may serve to—build in adopted principles of ancestry and population diversity, and in that context, the report by M-A Song et al., despite the exploratory study design, opens the door to descriptive methylation indices that is related to a more refined reference to ancestry [10]. An optimistic outlook on future projects, due consideration to statistical and power estimates that reach genome-wide significance, is warranted.

## Data Availability

Not applicable.
